# Effectiveness of recombinant zoster vaccine in reducing herpes zoster incidence and all-cause mortality among patients with rheumatoid arthritis: a retrospective cohort study of 21,046 individuals from TriNetX U.S. Collaborative Network

**DOI:** 10.1016/j.eclinm.2025.103319

**Published:** 2025-06-25

**Authors:** Ying-Li Lin, Shiow-Ing Wang, James Cheng-Chung Wei

**Affiliations:** a Department of Family Medicine, Changhua Christian Hospital, Changhua, Taiwan; b Institute of Medicine, Chung Shan Medical University, Taichung, Taiwan; c Department of Post-Baccalaureate Medicine, College of Medicine, National Chung Hsing University, Taichung, Taiwan; d Center for Health Data Science, Chung Shan Medical University Hospital, Taichung, Taiwan; e Department of Allergy, Immunology & Rheumatology, Chung Shan Medical University Hospital, Taichung, Taiwan; f Department of Nursing, Chung Shan Medical University, Taichung, Taiwan; g Graduate Institute of Integrated Medicine, China Medical University, Taichung, Taiwan; h Department of Medical Research, Taichung Veterans General Hospital, Taichung, Taiwan

**Keywords:** Rheumatoid arthritis, Herpes zoster, Recombinant zoster vaccine, Mortality

## Abstract

**Background:**

Patients with rheumatoid arthritis (RA) are at increased risk of developing herpes zoster (HZ). This study aimed to assess the effectiveness of recombinant zoster vaccine (RZV) in reducing both the incidence of HZ and all-cause mortality among individuals with RA.

**Methods:**

We conducted a retrospective cohort study using data from the TriNetX U.S. Collaborative Network between October 1, 2017 and March 31, 2024. The study population consisted of patients with RA who received RZV (RA-RZV cohort) and those who did not (RA control cohort). Propensity score matching (PSM) was employed to balance baseline characteristics between the groups, accounting for demographics, social economic status, lifestyles, medical utilization, comorbidities, and medications. The primary outcomes were the risk of HZ and all-cause mortality. PSM-adjusted hazard ratios (HRs) were calculated using Cox proportional hazards regression models. Kaplan–Meier curves and Log-Rank tests were used to evaluate survival differences.

**Findings:**

After PSM, each cohort included 21,046 individuals. The RA-RZV group demonstrated significantly lower risks of HZ (HR: 0.836, 95% CI: 0.738–0.947) and all-cause mortality (HR: 0.606, 95% CI: 0.561–0.654). The HZ risk reduction was more pronounced in patients aged 50–64 years (HR: 0.731, 95% CI: 0.610–0.876), females (HR: 0.791, 95% CI: 0.684–0.915), White (HR: 0.808, 95% CI: 0.696–0.937), and Black/African American participants (HR: 0.685; 95% CI: 0.481–0.976).

**Interpretation:**

RZV vaccination is associated with a reduced risk of HZ among patients with RA aged 50 years and older. While we observed a reduction in all-cause mortality among RZV recipients, this finding should be interpreted with caution, as the effect size may not be entirely attributable to HZ prevention alone. Given the established efficacy of RZV in preventing HZ and its potential to reduce mortality, vaccination should be prioritized among eligible patients with RA.

**Funding:**

There is no funding.

**Research in context**

**Evidence before this study**

Patients with rheumatoid arthritis (RA) are at increased risk for herpes zoster (HZ) infection. Although the recombinant zoster vaccine (RZV) is available, its effectiveness in individuals with RA remains insufficiently characterized. To identify existing evidence, we conducted a PubMed search covering the period from January 1, 2015 to June 30, 2024, using the terms “rheumatoid arthritis” and “recombinant zoster vaccine,” restricted to English-language publications. The search yielded six relevant studies, which primarily focused on the immunogenicity and safety of RZV in RA patients. However, none of the studies assessed the real-world effectiveness of RZV in this population.

**Added value of this study**

Using data from the TriNetX U.S. Collaborative Network, this large-scale retrospective cohort study demonstrates RZV's effectiveness in reducing both HZ incidence and all-cause mortality among patients with RA. The findings revealed significant reductions in both HZ incidence (16.4%) and all-cause mortality (39.4%). The protective effect against HZ was particularly pronounced in specific demographic subgroups, including patients aged 50–64 years, females, and White or Black/African American individuals.

**Implications of all the available evidence**

These findings provide robust evidence supporting RZV vaccination recommendations for patients with RA aged 50 and older. Future efforts should focus on developing and implementing targeted strategies to improve RZV vaccine uptake in this high-risk population.

## Introduction

Rheumatoid arthritis (RA) is a chronic, systemic autoimmune inflammatory disease primarily affecting peripheral joints symmetrically.^1^ Recent epidemiological data indicate a significant upward trend in both the prevalence and incidence of RA, with age-standardized prevalence increasing by 7.4% and incidence rising by 8.2% between 1990 and 2017.^2^ Patients with RA face an increased risk of serious infections compared to the general population, likely due to a combination of immunological dysfunction, comorbidities, and immunosuppressive therapies.^3^^,^^4^

Herpes zoster (HZ), caused by reactivation of latent varicella-zoster virus (VZV), is one such infection of particular concern. HZ typically manifests with prodromal neuralgia followed by unilateral vesicular rash along sensory nerve distributions.^5^ It can lead to significant long-term sequelae, including post-herpetic neuralgia, increased risk of cardiovascular events, and vision impairment related to herpes zoster ophthalmicus.^6^, ^7^, ^8^ The global incidence of HZ is rising, imposing substantial economic burdens on healthcare systems and society.^9^ Advanced age and immunosuppression are known risk factors for HZ.^10^ Notably, patients with RA have an estimated incidence rate ratio of 1.93 for developing HZ compared to those without RA,^11^ with certain RA treatments, such as Janus kinase inhibitors, conferring additional risk.^12^

HZ is one of the most prevalent vaccine-preventable diseases. The recombinant zoster vaccine (RZV; Shingrix; GlaxoSmithKline) was approved by the Food and Drug Administration (FDA) in October 2017 for adults aged 50 or older.^13^ Clinical trials demonstrated impressive efficacy, with RZV reducing HZ incidence by 97.2% in adults 50 and older (ZOE-50) and 89.8% in those 70 and older (ZOE-70).^14^^,^^15^ These results established RZV as the preferred vaccine for HZ prevention in immunocompetent adults. A subset analysis of ZOE-50/70 participants with pre-existing potential immune-mediated diseases showed that RZV was 90.5% efficacious against confirmed HZ cases.^16^ However, while small studies have demonstrated RZV's immunogenicity and acceptable safety profile in patients with RA on various therapies,^17^, ^18^, ^19^ real-world efficacy data in this population remain limited.

Given the increased risk of HZ in patients with RA and the potential for severe complications, there is an urgent need for studies on the real-world use of RZV in this population. To address this knowledge gap, we conducted a retrospective cohort study of patients with RA to evaluate the efficacy of RZV in reducing both HZ incidence and mortality, aiming to provide crucial insights to inform clinical practice.

## Methods

### Study design and data source

This retrospective cohort study utilized the TriNetX analytics platform, a web-based database containing deidentified electronic health records of over 100 million patients from multiple countries. The database encompasses a wide range of information, including demographic details, diagnoses (coded according to the *International Classification of Diseases, Tenth Revision, Clinical Modification* [*ICD-10-CM*] codes), medications (coded using RxNorm or Anatomical Therapeutic Chemical codes), procedures (coded according to the *International Classification of Diseases, Tenth Revision, Procedure Coding System* [*ICD-10-PCS*] or Current Procedural Terminology [CPT]), and laboratory measurements (coded using Logical Observation Identifiers Names and Codes). The study employed a retrospective cohort analysis using electronic health records extracted from the TriNetX U.S. Collaborative Network database, which included approximately 100 million patients. Data analysis was conducted in July 2024, with the study period confined to October 1, 2017 through March 31, 2024.

The TriNetX platform complies with both the Health Insurance Portability & Accountability Act and the General Data Protection Regulation. Due to the aggregated nature of the data and the provision of only statistical summaries of de-identified information, the Western Institutional Review Board has granted TriNetX a waiver of informed consent. This ethical approach balances research needs with privacy protection, as individual patient identification is not possible, and the retrospective analysis of anonymized data poses minimal risk to participants. This study received approval from the Institutional Review Board of Chung Shan Medical University Hospital (CSMUH No: CS2-21176).

This report adheres to the guidelines governing the reporting of studies conducted using routinely collected observational health data statements for cohort studies.

### Participants

**Fig. 1:**
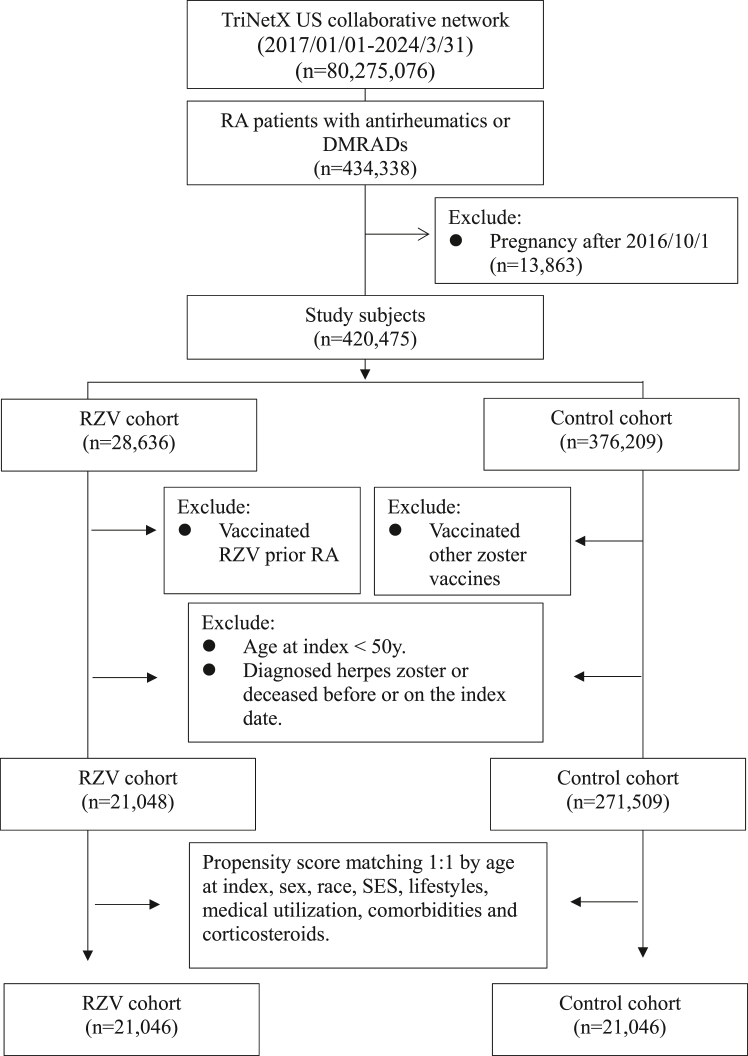
Selection process.

Study subjects comprised patients diagnosed with RA, identified through a diagnostic code for rheumatoid arthritis (ICD-10 = M05–M06) and a medication code indicating prescription of at least one of the following antirheumatic or disease-modifying antirheumatic drugs (DMARD): methotrexate, hydroxychloroquine, sulfasalazine, leflunomide, etanercept, adalimumab, tocilizumab, abatacept, golimumab, certolizumab pegol, infliximab, rituximab, azathioprine, secukinumab, ixekizumab, infliximab, guselkumab, cyclosporine, mycophenolate mofetil, ustekinumab, belimumab, tofacitinib or cyclophosphamide (Supplementary Table S1). To elucidate the effects of RZV in patients with RA, we excluded individuals, who became pregnant after October 1, 2016. The RA-RZV cohort included patients with RA who had either a CPT code for RZV administration (CPT: 90750) or a Rxnorm code for RZV. Patients who had received RZV before RA diagnosed were excluded. The RA control cohort included patients with RA who did not have a CPT code or Rxnorm code for RZV or the live zoster vaccine [Zostavax; Merck, Kenilworth, NJ]. All groups excluded subjects who were under 50 years of age, diagnosed with HZ at any time prior to the index date, or deceased before or on the index date. The index date was defined as the date of RZV administration for the RA-RZV cohort and the date of RA diagnosis for the control cohort. A flowchart of the selection process can be found in Fig. 1.

### Covariates

The following covariate factors (obtained from records spanning 1 year before the index date) were incorporated in this study to reduce confounding effects.

#### Demographics

Demographic factors of interest included age, sex, race, and social economic status (SES) encoded as a proxy code (ICD-10 = Z59, Persons with potential health hazards related to socioeconomic and psychosocial circumstances).

#### Lifestyles

Lifestyle plays an important role in the progression of diseases. The lifestyle variables were identified with the ICD-10 codes and matched in this study, including tobacco use (Z72.0, proxy smoking), nicotine dependence (F17, proxy smoking), and alcohol-related disorders (F10, proxy alcohol drinking).

#### Medical utilization

To balance the health status or medical utilization between groups, patients were matched on office or other outpatient services (CPT code 1013626), preventive medicine services (CPT code 1013829), emergency department services (CPT code 1013711) and hospital inpatient services (CPT code 1013659) characteristics.

#### Comorbidities

The comorbidities matched in the present study were identified with the ICD-10 codes, including essential hypertension (I10), hyperlipidemia (E78.5), diabetes mellitus (E8–E13), neoplasm (C00–D49), sleep disorders (G47), chronic lower respiratory diseases (J40–J47), vitamin D deficiency (E55), depressive episode (F32), ischemic heart diseases (I20–I25), chronic kidney disease (N18), liver diseases (K70–K77), cerebrovascular diseases (I60–I69), diseases of the blood and blood-forming organs and certain disorders involving the immune mechanism (D50–D89), vitamin B deficiency (E53), psoriasis (L40), non-infective enteritis and colitis (K50–K52), systemic lupus erythematosus (M32), ankylosing spondylitis (M45), and transplanted organ and tissue status (Z94).

#### Medications

Subjects were divided into medication users or non-users based on the prescription information. In the present study, subjects were matched on corticosteroids for systemic use (ATC: H02).

### Outcomes

The primary outcomes were the risk of HZ and all-cause mortality in the RA-RZV cohort compared to the RA control cohort. Patients who developed HZ were identified using ICD-10 codes for herpes zoster (B02). All-cause mortality was determined by a vital status of deceased. Secondary outcomes encompassed the risk of specific HZ manifestations, categorized using the following ICD-10 codes: zoster encephalitis (B02.0), zoster meningitis (B02.1), zoster with other nervous system involvement (B02.2), zoster ocular disease (B02.3), disseminated zoster (B02.7), zoster with other complications (B02.8), and zoster without complications (B02.9).

### Statistical analyses

All statistical analyses were conducted using the TriNetX platform. To reduce the effect of confounding factors, propensity score matching (PSM) at a 1:1 ratio was performed to balance the following covariates between the RA-RZV and RA control groups: age at index, sex, race, SES, lifestyle-related proxy variables, medical utilization, comorbidities, and medications. The balance of baseline characteristics between matched cohorts was assessed using standardized mean differences (SMDs). Variables with a SMD less than 0.1 were considered well-matched. Cox proportional hazards regression analysis was employed to compare the matched cohorts, providing hazard ratios (HRs) coupled with 95% confidence intervals (CIs). Due to inherent limitations of the TriNetX platform, only Kaplan–Meier curves representing observed survival after propensity score matching are available, while the system cannot generate adjusted time-to-event curves from the Cox regression models. The Kaplan–Meier analysis was used to estimate the probability of the outcome of interest, and the log-rank test assessed differences in survival curves between groups. A two-sided p-value of <0.05 indicated statistical significance.

Six sensitivity analyses were conducted to confirm the robustness of our results. First, we calculated the E-value to quantitatively assess the minimum strength of residual confounding required to nullify the observed association in the primary analysis.^20^ Second, we examined the temporal effects of RZV by analyzing effectiveness across distinct periods: 1 day–1 year, 1 day–2 years, 1 day–3 years, and 1 day–4 years post-vaccination. Third, we employed propensity score matching with various covariate combinations to evaluate the consistency of RZV effectiveness. Fourth, to ensure accurate RA patient identification, we performed an analysis excluding participants who had received treatments not indicated for RA (including secukinumab, ixekizumab, guselkumab, mycophenolate mofetil, ustekinumab, and belimumab). Fifth, we investigated the differential effectiveness between individuals who completed the two-dose RZV series and those who received only a single dose. Finally, we compared the effectiveness of RZV between patients with RA and individuals without RA to assess the specific impact of RA on the vaccine response.

Subgroup analyses based on age at index, sex, and race were also performed to explore the difference between these groups.

### Patient and public involvement

The data utilized were deidentified and obtained from the TriNetX U.S. Collaborative Network, which precluded any direct contact with the individuals whose data were analyzed. Due to the nature of this secondary data analysis and the restrictions imposed by the data source, it was neither permissible nor feasible to engage patients or members of the public in the study design, conduct, or dissemination of results. While we acknowledge the value of patient and public involvement in research, the constraints of our data source made such involvement impractical in this instance.

### Role of the funding source

There was no funding source for this study.

## Results

### Characteristics of the study subjects

Our study design identified 21,048 patients in the RA-RZV cohort and 271,509 in the RA control cohort during the study period. After PSM, we included 21,046 RZV-vaccinated patients and an equal number of non-vaccinated patients. The selection process is illustrated in Fig. 1.

Table 1 presents the baseline characteristics of the RA-RZV and RA control cohorts before and after PSM, including demographics, SES, lifestyles, medical utilization, comorbidities, and medications. In the RA-RZV group, the mean age was 64.8 ± 8.8 years, with 70.9% of participants being female and the majority (68.8%) identifying as White. Before matching, the groups showed differences in medical utilization, comorbidities, and medications. After matching, these differences fell within the acceptable range (SMD < 0.1).

**Table 1:** Baseline characteristics of study subjects (before and after PSM matching).

Variables	Before PSM^a^			After PSM^a^		
	RZV cohort (n = 21,048)	Control cohort (n = 271,509)	SMD	RZV cohort (n = 21,046)	Control cohort (n = 21,046)	SMD
**Age at index, y**						
Mean ± SD	64.8 ± 8.8	65.4 ± 9.5	0.065	64.8 ± 8.8	64.9 ± 9.2	0.019
**Sex, n (%)**						
Female	14,926 (70.9)	190,246 (70.1)	0.019	14,925 (70.9)	14,995 (71.2)	0.007
Male	4966 (23.6)	67,391 (24.8)	0.029	4965 (23.6)	4950 (23.5)	0.002
Unknown Gender	1156 (5.5)	13,872 (5.1)	0.017	1156 (5.5)	1101 (5.2)	0.012
**Race, n (%)**						
White	14,481 (68.8)	185,170 (68.2)	0.013	14,479 (68.8)	14,790 (70.3)	0.032
Black or African American	2968 (14.1)	34,684 (12.8)	0.039	2968 (14.1)	2868 (13.6)	0.014
Asian	605 (2.9)	6338 (2.3)	0.034	605 (2.9)	589 (2.8)	0.005
Other race	766 (3.6)	9068 (3.3)	0.016	766 (3.6)	708 (3.4)	0.015
American Indian or Alaska native	79 (0.4)	886 (0.3)	0.008	79 (0.4)	69 (0.3)	0.008
Native Hawaiian or other Pacific Islander	49 (0.2)	530 (0.2)	0.008	49 (0.2)	36 (0.2)	0.014
Unknown race	2100 (10.0)	34,833 (12.8)	0.090	2100 (10.0)	1986 (9.4)	0.018
**Social economic status, n (%)**						
Problems related to housing and economic circumstances	150 (0.7)	1302 (0.5)	0.030	150 (0.7)	156 (0.7)	0.003
**Lifestyles, n (%)**						
Nicotine dependence	2001 (9.5)	25,460 (9.4)	0.004	2001 (9.5)	1934 (9.2)	0.011
Tobacco use	729 (3.5)	6264 (2.3)	0.069	728 (3.5)	712 (3.4)	0.004
Alcohol related disorders	406 (1.9)	4732 (1.7)	0.014	405 (1.9)	360 (1.7)	0.016
**Medical utilization, n (%)**						
Office or other outpatient services	17,310 (82.2)	161,740 (59.6)	**0.515**	17,308 (82.2)	17,527 (83.3)	0.028
Preventive medicine services	4963 (23.6)	13,632 (5.0)	**0.550**	4961 (23.6)	4757 (22.6)	0.023
Emergency department services	4203 (20.0)	60,640 (22.3)	0.058	4202 (20.0)	3904 (18.6)	0.036
Hospital inpatient and observation care services	2048 (9.7)	32,339 (11.9)	0.070	2048 (9.7)	1934 (9.2)	0.019
**Comorbidities, n (%)**						
Essential (primary) hypertension	12,064 (57.3)	125,348 (46.2)	**0.225**	12,062 (57.3)	12,129 (57.6)	0.006
Hyperlipidemia, unspecified	7033 (33.4)	68,093 (25.1)	**0.184**	7031 (33.4)	7119 (33.8)	0.009
Diseases of the blood and blood-forming organs and certain disorders involving the immune mechanism	6586 (31.3)	70,459 (26.0)	**0.118**	6584 (31.3)	6533 (31.0)	0.005
Neoplasms	5350 (25.4)	48,955 (18.0)	**0.180**	5348 (25.4)	5522 (26.2)	0.019
Sleep disorders	4951 (23.5)	42,312 (15.6)	**0.201**	4949 (23.5)	4923 (23.4)	0.003
Chronic lower respiratory diseases	4965 (23.6)	55,732 (20.5)	0.074	4965 (23.6)	4875 (23.2)	0.010
Diabetes mellitus	4773 (22.7)	53,580 (19.7)	0.072	4772 (22.7)	4667 (22.2)	0.012
Vitamin D deficiency	4594 (21.8)	30,657 (11.3)	**0.286**	4592 (21.8)	4488 (21.3)	0.012
Depressive episode	4025 (19.1)	37,370 (13.8)	**0.145**	4023 (19.1)	3903 (18.5)	0.015
Ischemic heart diseases	3081 (14.6)	39,528 (14.6)	0.002	3080 (14.6)	3045 (14.5)	0.005
Chronic kidney disease (CKD)	2307 (11.0)	24,898 (9.2)	0.060	2305 (11.0)	2269 (10.8)	0.005
Diseases of liver	1588 (7.5)	16,275 (6.0)	0.062	1588 (7.5)	1509 (7.2)	0.014
Cerebrovascular diseases	1318 (6.3)	16,484 (6.1)	0.008	1318 (6.3)	1289 (6.1)	0.006
Deficiency of other B group vitamins	1197 (5.7)	7518 (2.8)	**0.145**	1196 (5.7)	1139 (5.4)	0.012
Psoriasis	1173 (5.6)	9719 (3.6)	0.095	1172 (5.6)	1114 (5.3)	0.012
Noninfective enteritis and colitis	1137 (5.4)	10,921 (4.0)	0.065	1137 (5.4)	1109 (5.3)	0.006
Systemic lupus erythematosus (SLE)	768 (3.6)	9773 (3.6)	0.003	768 (3.6)	701 (3.3)	0.017
Ankylosing spondylitis	270 (1.3)	2535 (0.9)	0.033	270 (1.3)	276 (1.3)	0.003
Transplanted organ and tissue status	251 (1.2)	2429 (0.9)	0.029	250 (1.2)	250 (1.2)	0.000
**Comedication, n (%)**						
Corticosteroids for systemic use	12,755 (60.6)	152,025 (56.0)	0.094	12,753 (60.6)	12,641 (60.1)	0.011
NSAIDs	10,080 (47.9)	147,193 (54.2)	**0.127**	10,080 (47.9)	11,765 (55.9)	**0.161**
Other DMARDs						
Methotrexate	5465 (26.0)	61,198 (22.5)	0.080	5465 (26.0)	4446 (21.1)	**0.114**
Hydroxychloroquine	4347 (20.7)	52,853 (19.5)	0.030	4347 (20.7)	4035 (19.2)	0.037
Leflunomide	1503 (7.1)	15,755 (5.8)	0.054	1503 (7.1)	1182 (5.6)	0.062
Adalimumab	1476 (7.0)	11,938 (4.4)	**0.113**	1476 (7.0)	913 (4.3)	**0.116**
Sulfasalazine	1187 (5.6)	11,299 (4.2)	0.069	1187 (5.6)	936 (4.4)	0.055
Etanercept	1066 (5.1)	9310 (3.4)	0.081	1066 (5.1)	754 (3.6)	0.073
Tofacitinib	830 (3.9)	5278 (1.9)	**0.118**	830 (3.9)	390 (1.9)	**0.125**
Abatacept	568 (2.7)	4778 (1.8)	0.064	568 (2.7)	341 (1.6)	0.074
Rituximab	472 (2.2)	3416 (1.3)	0.075	472 (2.2)	255 (1.2)	0.079
Infliximab	393 (1.9)	3614 (1.3)	0.043	393 (1.9)	281 (1.3)	0.042
Tocilizumab	345 (1.6)	2640 (1.0)	0.059	345 (1.6)	194 (0.9)	0.064
Azathioprine	257 (1.2)	3554 (1.3)	0.008	257 (1.2)	297 (1.4)	0.017
Upadacitinib	248 (1.2)	1276 (0.5)	0.078	248 (1.2)	77 (0.4)	0.093
Golimumab	190 (0.9)	1802 (0.7)	0.027	190 (0.9)	130 (0.6)	0.033
Certolizumab pegol	126 (0.6)	1346 (0.5)	0.014	126 (0.6)	112 (0.5)	0.009
Apremilast	89 (0.4)	702 (0.3)	0.028	89 (0.4)	81 (0.4)	0.006
Secukinumab	85 (0.4)	621 (0.2)	0.031	85 (0.4)	62 (0.3)	0.019
Ustekinumab	70 (0.3)	424 (0.2)	0.036	70 (0.3)	49 (0.2)	0.019
Sarilumab	66 (0.3)	306 (0.1)	0.044	66 (0.3)	16 (0.1)	0.054
Ixekizumab	33 (0.2)	236 (0.1)	0.020	33 (0.2)	19 (0.1)	0.019

PSM: propensity score matching, RZV: Recombinant zoster vaccine, SMD: standardized mean difference, SD: standard deviation, DMARDs: disease-modifying antirheumatic drugs, NSAIDs: Anti-inflammatory and antirheumatic products, non-steroids.

If the patient number is less than or equal to 10, the results show the count as 10.

Bold font represents a standardized difference greater than 0.1.

a Propensity score matching was performed on age at index, sex, race, SES, lifestyles, medical utilization, comorbidities, and corticosteroids.

### Risk of HZ and all-cause mortality after RZV

**Fig. 2:**
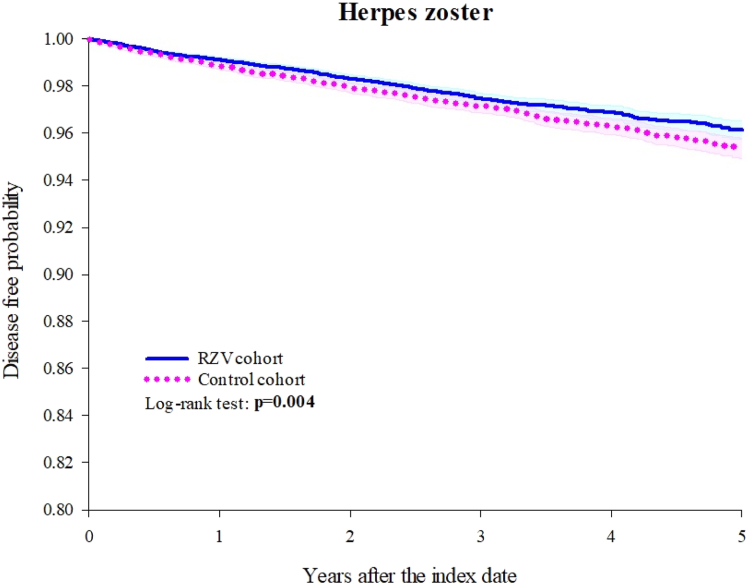
Kaplan–Meier curve of herpes zoster.

**Fig. 3:**
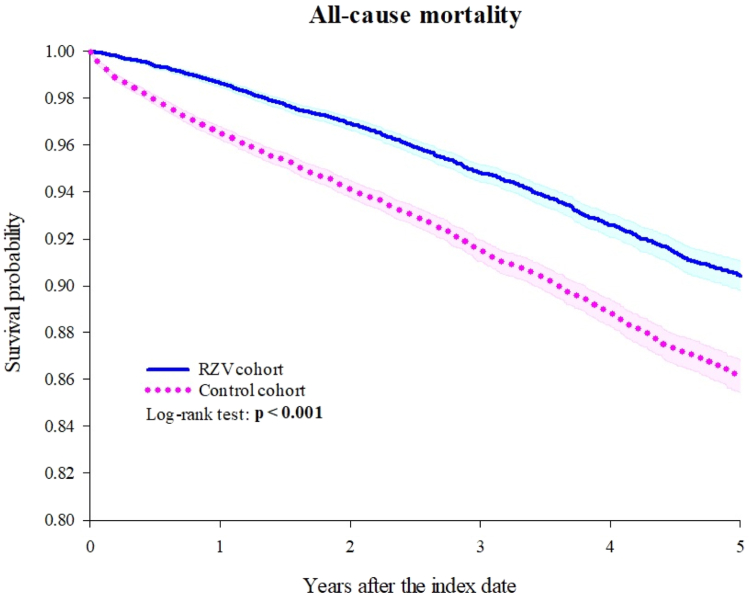
Kaplan–Meier curve of all-cause mortality.

Table 2 presents the HR (95% CI) for outcomes in the compared groups. The RA-RZV cohort exhibited a lower risk of HZ compared to the RA control cohort, with an HR of 0.836 (95% CI: 0.738–0.947). The Kaplan–Meier curves (Fig. 2) demonstrate a significant difference in HZ risk between the RA-RZV and RA control cohorts (log-rank test, p = 0.004). Furthermore, the RA-RZV cohort showed a significantly lower all-cause mortality risk compared to the RA control cohort (HR: 0.606, 95% CI: 0.561–0.654). The Kaplan–Meier curves (Fig. 3) illustrate a significant difference in mortality risk between the two cohorts (log-rank test, p < 0.001).

**Table 2:** Risk of outcomes (1 day–5 years).

Outcomes	Patients with outcome		Hazard ratio (95% CI)^a^	E-value for point estimate (E-value for the CI)
	RZV cohort (n = 21,046)	Control cohort (n = 21,046)		
**Herpes zoster**	475	525	**0.836** (**0.738–0.947)**	**1.68** (**1.30)**
Zoster encephalitis	10	10	0.691 (0.155–3.086)	2.25 (1.00)
Zoster meningitis	10	10	0.919 (0.057–14.69)	1.40 (1.00)
Zoster with other nervous system involvement	124	128	0.890 (0.696–1.140)	1.50 (1.00)
Zoster ocular disease	31	45	0.646 (0.409–1.021)	2.47 (1.00)
Disseminated zoster	12	14	0.795 (0.367–1.719)	1.83 (1.00)
Zoster with other complications	61	69	0.811 (0.575–1.145)	1.77 (1.00)
Zoster without complications	377	389	0.898 (0.779–1.035)	1.47 (1.00)
**All-cause mortality**	1069	1642	**0.606** (**0.561–0.654)**^b^	**2.69** (**2.43)**

RZV: Recombinant zoster vaccine, CI: Confidence interval, NA: Not available.

If the patient is less or equal to 10, results show the count as 10.

a Propensity score matching was performed on age at index, sex, race, SES, lifestyles, medical utilization, comorbidities, and corticosteroids.

b Proportionality < 0.001.

### Risk of specific HZ manifestations

No significant differences were observed between the RA-RZV and RA control cohorts regarding the risk of specific HZ manifestations (Table 2). This included zoster encephalitis (HR: 0.691, 95% CI: 0.155–3.086), zoster meningitis (HR: 0.919, 95% CI: 0.057–14.69), zoster with other nervous system involvement (HR: 0.890, 95% CI: 0.696–1.140), zoster ocular disease (HR: 0.646, 95% CI: 0.409–1.021), disseminated zoster (HR: 0.795, 95% CI: 0.367–1.719), zoster with other complications (HR: 0.811, 95% CI: 0.575–1.145), and zoster without complications (HR: 0.898, 95% CI: 0.779–1.035).

### Sensitivity analyses

We conducted six sensitivity analyses to evaluate the robust of results. The E-value was 1.68 (CI: 1.30) for herpes zoster and 2.69 (CI: 2.43) for all-cause mortality, indicating that any unmeasured confounder would need an association strength of ≥1.68 with both RZV vaccination and herpes zoster risk to nullify the observed protective effect of RZV against herpes zoster. Similarly, an unmeasured confounder would need association strength of ≥2.69 with both RZV vaccination and mortality risk to nullify the observed protective association between RZV vaccination and all-cause mortality in our primary analyses (Table 2). Supplementary Table S2 presents the comparative risks of outcomes between the RA-RZV and RA control groups across multiple follow-up intervals. The HR for HZ remained consistently lower in the vaccinated group across all time periods: 1 day–1 year (HR: 0.803, 95% CI: 0.657–0.982), 1 day–2 years (HR: 0.817, 95% CI: 0.699–0.954), 1 day–3 years (HR: 0.861, 95% CI: 0.750–0.989), and 1 day–4 years (HR: 0.838, 95% CI: 0.736–0.954). Similarly, the HR for all-cause mortality was substantially lower in the vaccinated group across all follow-up periods: 1 day–1 year (HR: 0.375, 95% CI: 0.324–0.433), 1 day–2 years (HR: 0.493, 95% CI: 0.444–0.548), 1 day–3 years (HR: 0.559, 95% CI: 0.511–0.611), and 1 day–4 years (HR: 0.593, 95% CI: 0.547–0.643). While the protective effect against all-cause mortality gradually attenuated with longer follow-up durations, the RA-RZV group consistently demonstrated significantly lower risks for both herpes zoster and all-cause mortality compared to the RA control group throughout all examined time frames.

Another sensitivity analysis employing propensity score matching with varied covariate combinations (four distinct models incorporating all listed variables) yielded consistent results across Models 3 and 4, confirming that the RA-RZV group maintained significantly lower risks for both herpes zoster and all-cause mortality compared to the RA control group (Supplementary Table S3). Additionally, a sensitivity analysis to ensure accurate RA patient identification by excluding participants who had received treatments not indicated for RA (including secukinumab, ixekizumab, guselkumab, mycophenolate mofetil, ustekinumab, and belimumab) similarly demonstrated that RZV vaccination was associated with reduced risks of herpes zoster (HR: 0.873, 95% CI: 0.784–0.973) and all-cause mortality (HR: 0.623, 95% CI: 0.582–0.666) (Supplementary Table S4).

Supplementary Table S5 demonstrates that patients with RA who completed the two-dose RZV series exhibited significantly lower risks of zoster with complications (HR: 0.481, 95% CI: 0.271–0.855) and all-cause mortality (HR: 0.635, 95% CI: 0.565–0.715) compared to those who received only a single dose. However, the risk of overall HZ did not differ significantly between the two-dose and single-dose vaccination groups. In our final sensitivity analysis comparing RZV effectiveness between patients with RA and individuals without RA, Supplementary Table S6 revealed that patients with RA demonstrated a higher risk of herpes zoster (HR: 2.033, 95% CI: 1.765–2.342) but a lower risk of all-cause mortality (HR: 0.853, 95% CI: 0.793–0.918) compared to individuals without RA.

### Subgroup analyses

In our stratified analysis, we examined the risk of outcomes across subgroups defined by age, sex, and race. Among individuals aged 50–64 years, the RA-RZV group demonstrated a significant lower risk of HZ (HR: 0.731, 95% CI: 0.610–0.876). This group also exhibited a reduced risk of all-cause mortality in both the 50–64 years subgroup (HR: 0.578, 95% CI: 0.501–0.668) and the ≥65 years subgroup (HR: 0.637, 95% CI: 0.581–0.698) (Supplementary Table S7).

Sex-stratified analysis revealed that the RA-RZV group had a lower risk of HZ among female (HR: 0.791, 95% CI: 0.684–0.915). Moreover, this group showed a decreased risk of all-cause mortality in both males (HR: 0.601, 95% CI: 0.524–0.689) and females (HR: 0.632, 95% CI: 0.572–0.698) (Supplementary Table S8).

In the race-stratified analysis, the RA-RZV group demonstrated a lower risk of HZ in both the White subgroup (HR: 0.808, 95% CI: 0.696–0.937) and the Black/African American subgroup (HR: 0.685, 95% CI: 0.481–0.976). Similarly, this group exhibited a reduced risk of all-cause mortality among both White participants (HR: 0.642, 95% CI: 0.587–0.703) and Black/African American participants (HR: 0.496, 95% CI: 0.394–0.624) (Supplementary Table S9).

## Discussion

In this pioneering real-world assessment of RZV effectiveness among patients with RA, our findings demonstrate that vaccination significantly reduces both HZ risk and all-cause mortality compared to unvaccinated counterparts. These beneficial effects persisted across various sensitivity analyses, highlighting RZV's potential as a crucial preventive intervention for this immunologically vulnerable population.

Previous studies on RZV in patients with RA have primarily focused on immunogenicity and safety.^17^, ^18^, ^19^ A prospective study showed that RZV immunogenicity is not impaired in patients with RA receiving Janus kinase inhibitors (JAKis) or anti-cellular DMARDs.^17^ Another study demonstrated that RZV is immunogenic and has a clinically acceptable safety profile in patients with RA aged ≥50 years receiving DMARDs.^19^ Additionally, 80.5% of patients with RA treated with JAKi achieved a ≥4-fold increase in antibody levels following two-dose RZV vaccination.^18^ Our study extends this knowledge base by exploring RZV's real-world effectiveness in patients with RA.

The incidence of HZ is notably elevated in patients with RA.^11^ Advanced age, accompanied by the natural decline in immune function, alongside immunosuppression resulting from disease pathology or therapeutic interventions, are associated with an increased risk of HZ.^10^^,^^21^ RZV has been demonstrated to be immunogenic and effective in preventing HZ and its related complications. It also demonstrates an acceptable safety profile in adults aged 50 years and older in various settings, as well as in diverse immunocompromised patient populations aged 18 years and older.^22^^,^^23^ Our findings substantiate that RZV significantly reduces the 16.4% risk of HZ among vaccinated patients with RA when compared to their unvaccinated counterparts.

Clinical trials have demonstrated that RZV significantly reduces the incidence of herpes zoster (HZ), with efficacy rates of 97.2% in adults aged ≥50 years (ZOE-50) and 89.8% in those aged ≥70 years (ZOE-70).^14^^,^^15^ In real-world settings, RZV has shown effectiveness ranging from 70 to 86% against HZ among adults aged ≥50 years and 57–68% in patients with autoimmune diseases.^24^ While Izurieta et al. reported higher RZV efficacy in patients with autoimmune conditions, their study population was heterogeneous, encompassing multiple sclerosis, psoriasis, psoriatic arthritis, rheumatoid arthritis, systemic lupus erythematosus, and ulcerative colitis.^25^ In contrast, our study specifically focused on patients with RA receiving antirheumatic drugs or disease-modifying antirheumatic drugs (DMARDs), which may impair immune responses to vaccination.^26^^,^^27^ Our sensitivity analysis indicated that HZ risk remained elevated in RZV-vaccinated patients with RA compared to RZV-vaccinated individuals without RA, suggesting that the protective benefits of RZV may be attenuated in this population. Nonetheless, despite the potential immunosuppressive effects of underlying RA and its treatments on vaccine-induced immunity, our findings support the effectiveness of RZV in reducing HZ risk in this vulnerable group.

Previous studies have shown that patients with RA have an increased risk of mortality compared to the general population, with leading causes of death including cardiovascular disease, malignancies, respiratory diseases, and infections.^28^^,^^29^ In our study, patients with RA who received the RZV exhibited a lower risk of all-cause mortality compared to their unvaccinated counterparts. Sensitivity analyses further demonstrated that patients with RA who completed the two-dose RZV series had significantly lower risks of all-cause mortality than those who received a single dose. Notably, when assessing the relative benefit of vaccination, our sensitivity analysis indicated that patients with RA experienced a greater reduction in all-cause mortality risk following RZV vaccination compared to non-RA individuals. These findings suggest that this high-risk population may derive particular benefit from RZV immunization.

Three published studies have examined the association between RZV and mortality. One TriNetX-based study in the general population found that RZV was associated with reduced risks of myocardial infarction and overall mortality^30^; another study among patients with diabetes showed a reduction in major adverse cardiovascular events (MACE) and all-cause mortality following herpes zoster vaccination^31^; and a third study in individuals with immune-mediated inflammatory diseases treated with JAKi reported reduced all-cause mortality after RZV vaccination.^32^ Although these studies used TriNetX data, which limits the ability to determine cause-specific mortality, they consistently suggest a protective effect of RZV across different populations.

An intriguing finding from our study (Supplementary Table S2) was that the lowest all-cause mortality risk occurred within the first year after vaccination, followed by a slight increase over time, yet remaining significantly lower than in unvaccinated individuals throughout a four-year follow-up. This temporal pattern is consistent with a prior study showing that the strongest protective effect of HZ vaccination on MACE in diabetic patients was observed during the first year.^31^ Several mechanisms may explain this observation. One possibility is that RZV modulates the immune response and reduces systemic inflammation, a key driver of both cardiovascular and all-cause mortality.^31^^,^^33^ However, this remains speculative, as current evidence does not conclusively establish an immunomodulatory effect of RZV beyond its role in HZ prevention. Alternatively, the reduction in mortality risk may reflect RZV's prevention of HZ-related complications or its potential role in lowering cardiovascular risk, given the known association between HZ infection and increased risks of stroke and myocardial infarction, particularly within the first year after infection.^34^^,^^35^ Further research is needed to clarify the biological mechanisms underlying this temporal trend.

While our findings suggest a potential mortality benefit of RZV in patients with RA, they should be interpreted with caution. The observed reduction may be influenced by immortal time bias, as individuals must survive long enough to receive the vaccine. Additionally, confounding by health behaviors may play a role—patients who seek preventive care, such as vaccination, may have higher health literacy, better access to healthcare, and greater adherence to medical advice. Although our analyses adjusted for multiple known confounders, unmeasured variables may contribute to the observed effect, underscoring the need for careful interpretation.

The impact of RZV on the risk of HZ in patients with RA may vary across age and sex subgroups; however, relevant literature on this topic is limited. Our study found that vaccinated patients aged 50–64 years and females exhibited a significantly lower risk of HZ. This aligns with previous research indicating that females typically generate more robust antibody responses to vaccination compared to males.^36^ Moreover, a study on the public health impact of HZ vaccination in Hong Kong projected the greatest reduction in HZ cases with RZV in the youngest cohort, aged 50–59 years.^37^ The lack of sufficient data on age and sex subgroups in the current literature may have limited our ability to fully consider these factors in our analysis. Further research is required to comprehensively elucidate the impact of RZV on the risk of HZ across different age and sex subgroups in patients with RA.

This study has several notable strengths. First, leveraging the TriNetX platform, we analyzed a large cohort of patients with RA who received RZV, providing substantial statistical power. Second, we employed propensity score matching to mitigate the impact of confounding variables, despite the retrospective nature of the study. This approach allowed us to assess the effectiveness of RZV in patients with RA over an extended follow-up period. Third, we present novel findings on the risk of HZ and mortality after RZV vaccination in patients with RA. The consistency of our results across various sensitivity analyses underscores the robustness of our study findings.

Our study has several limitations. First, the retrospective nature of data collection precludes establishing causal relationships between RZV vaccination and the observed outcomes. Secondly, electronic health records used in this study have inherent limitations. Although we employed validated definitions and propensity score matching to mitigate bias, the potential for misclassification bias and residual confounding cannot be entirely eliminated. Third, the utilization of TriNetX data restricted our ability to analyze specific causes of death, which significantly limits our understanding of mortality patterns. Additionally, patients may have been lost to follow-up during the study period, potentially affecting our research findings and introducing bias in outcome assessments. This limitation should be considered when interpreting the results of our analysis. Fourth, while data were extracted from TriNetX U.S. Collaborative Network, all of the individuals were US residents, potentially limiting the generalizability of our findings to other ethnicities and healthcare systems. Lastly, we were unable to assess the effectiveness of RZV in patients with RA aged 18–49 years due to the small cohort size, likely resulting from the recent recommendation of RZV in this population and low vaccine uptake.^38^^,^^39^

In conclusion, our study suggests that RZV vaccination is associated with a reduced risk of HZ among patients with RA aged 50 years and older. While we observed a reduction in all-cause mortality among RZV recipients, this finding should be interpreted with caution, as the effect size may not be entirely attributable to HZ prevention alone. Further research incorporating detailed individual-level clinical data—including cause of death and serious complications such as myocardial infarction and stroke—is needed to better understand this association. Nonetheless, given RZV's established efficacy in preventing HZ and its potential benefits in reducing all-cause mortality, healthcare providers should prioritize patient education and promote vaccination among eligible populations with RA.

## Contributors

YL-Lin wrote the draft of the manuscript; SI-Wang and CC-Wei accessed and verified the underlying data; SI-Wang performed data analysis; CC-Wei revised the manuscript; CC-Wei designed and supervised the study. All the authors contributed to the revision of the manuscript and read and approved the submitted version.

## Data sharing statement

Data that support the findings of this study are available at http://trinetx.com (TriNetX Analytics Network).

## Declaration of interests

The authors declare no competing interests.

## Appendix A Supplementary data

Supplementary Tables S1–S9

Captions for Supplementary Materials
