# Supplementary material for: Effectiveness of recombinant zoster vaccine in reducing herpes zoster incidence and all-cause mortality among patients with rheumatoid arthritis: a retrospective cohort study of 21,046 individuals from TriNetX U.S. Collaborative Network
Source: eClinicalMedicine. 2025 Jun 25;85:103319. doi: 10.1016/j.eclinm.2025.103319 (PMC12246860; doi:10.1016/j.eclinm.2025.103319)
Supplement: Supplementary Tables S1–S9 [file mmc1.docx]

**Supplementary Table 1.** Codes utilized in cohort identification

| RA medications | Codes |
| --- | --- |
| ANTIRHEUMATICS | NLM:VA:MS100 |
| methotrexate | NLM:RXNORM:6851 |
| hydroxychloroquine | NLM:RXNORM:5521 |
| sulfasalazine | NLM:RXNORM:9524 |
| leflunomide | NLM:RXNORM:27169 |
| etanercept | NLM:RXNORM:214555 |
| adalimumab | NLM:RXNORM:327361 |
| tocilizumab | NLM:RXNORM:612865 |
| abatacept | NLM:RXNORM:614391 |
| golimumab | NLM:RXNORM:819300 |
| certolizumab pegol | NLM:RXNORM:709271 |
| infliximab | NLM:RXNORM:191831 |
| rituximab | NLM:RXNORM:121191 |
| azathioprine | NLM:RXNORM:1256 |
| secukinumab | NLM:RXNORM:1599788 |
| ixekizumab | NLM:RXNORM:1745099 |
| infliximab | NLM:RXNORM:191831 |
| guselkumab | NLM:RXNORM:1928588 |
| cyclosporine | NLM:RXNORM:3008 |
| mycophenolate mofetil | NLM:RXNORM:68149 |
| ustekinumab | NLM:RXNORM:847083 |
| belimumab | NLM:RXNORM:1092437 |
| tofacitinib | NLM:RXNORM:1357536 |
| cyclophosphamide | NLM:RXNORM:3002 |

Note:

NLM: National Library of Medicine

VA: Veterans Affairs Drug Classification system

RXNORM: Normalized naming system for generic and branded drugs

**Supplementary Table 2**. Risk of outcomes_ different follow up duration

| **Outcomes**  **(**RZV cohort vs. Control cohort) | **Hazard ratio (95% CI)^a^** | | | |
| --- | --- | --- | --- | --- |
|  | 1 day to 1 year | 1 day to 2 years | 1 day to 3 years | 1 days to 4 years |
| **Herpes zoster** | **0.803 (0.657-0.982)** | **0.817 (0.699-0.954)** | **0.861 (0.750-0.989)** | **0.838 (0.736-0.954)** |
| Zoster encephalitis | 0.461 (0.084-2.517) | 0.461 (0.084-2.517) | 0.461 (0.084-2.517) | 0.691 (0.155-3.086) |
| Zoster meningitis | NA | NA | 0.919 (0.057-14.69) | 0.919 (0.057-14.69) |
| Zoster with nervous system involvement | 0.785 (0.530-1.164) | 0.917 (0.674-1.249) | 0.938 (0.716-1.229) | 0.948 (0.735-1.223) |
| Zoster ocular disease | 0.492 (0.219-1.104) | 0.595 (0.323-1.096) | **0.568 (0.333-0.969)** | 0.616 (0.378-1.003) |
| Disseminated zoster | 0.793 (0.266-2.360) | 0.913 (0.362-2.300) | 0.835 (0.368-1.892) | 0.846 (0.386-1.854) |
| Zoster with other complications | **0.461 (0.258-0.826)** | 0.691 (0.456-1.049) | 0.733 (0.501-1.074) | 0.795 (0.557-1.135) |
| Zoster without complications | 0.900 (0.712-1.138) | 0.899 (0.751-1.076) | 0.962 (0.821-1.127) | 0.897 (0.773-1.040) |
| **All-cause mortality** | **0.375 (0.324-0.433)^*^** | **0.493 (0.444-0.548)^*^** | **0.559 (0.511-0.611)^*^** | **0.593 (0.547-0.643)^*^** |

Note:

RZV: Recombinant zoster vaccine, CI: Confidence interval. NA: Not available.

a. Propensity score matching was performed on age at index, sex, race, SES, lifestyles, medical utilization, comorbidities and corticosteroids.

* Proportionality <0.001.

**Supplementary Table 3**. Risk of outcomes_ matching with different variables

| **Outcomes**  **(**RZV cohort vs. Control cohort) | **Hazard ratio (95% CI)** | | | |
| --- | --- | --- | --- | --- |
|  | Model 1 ^a^ | Model 2 ^b^ | Model 3 ^c^  (Main model) | Model 4 ^d^ |
| **Herpes zoster** | 1.050 (0.956-1.154) | 0.918 (0.809-1.042) | **0.836 (0.738-0.947)** | **0.881 (0.789-0.985)** |
| Zoster encephalitis | 1.130 (0.344-3.710) | 2.798 (0.291-26.90) | 0.691 (0.155-3.086) | 0.892 (0.180-4.423) |
| Zoster meningitis | 0.797 (0.105-6.061) | 0.990 (0.062-15.83) | 0.919 (0.057-14.69) | 0.903 (0.056-14.45) |
| Zoster with nervous system involvement | 1.134 (0.942-1.364) | 1.025 (0.794-1.324) | 0.890 (0.696-1.140) | 0.942 (0.745-1.191) |
| Zoster ocular disease | 0.919 (0.637-1.325) | 0.726 (0.454-1.160) | 0.646 (0.409-1.021) | 0.717 (0.456-1.127) |
| Disseminated zoster | 0.929 (0.516-1.675) | 0.922 (0.414-2.052) | 0.795 (0.367-1.719) | 0.948 (0.469-1.918) |
| Zoster with other complications | 1.013 (0.780-1.317) | 0.858 (0.606-1.216) | 0.811 (0.575-1.145) | 0.983 (0.727-1.330) |
| Zoster without complications | 1.097 (0.986-1.219) | 0.922 (0.800-1.064) | 0.898 (0.779-1.035) | 0.908 (0.801-1.028) |
| **All-cause mortality** | **0.551 (0.519-0.586)^*^** | **0.658 (0.609-0.712)^*^** | **0.606 (0.561-0.654)^*^** | **0.605 (0.566-0.648)^*^** |

Note:

RZV: Recombinant zoster vaccine, CI: Confidence interval. NA: Not available.

a. Crude, before matching.

b. Propensity score matching was performed on age at index, sex, race, SES, lifestyles, and medical utilization.

c. Propensity score matching was performed on age at index, sex, race, SES, lifestyles, medical utilization, comorbidities and corticosteroids.

d. Propensity score matching was performed on all listed variables.

* Proportionality <0.001.

**Supplementary Table 4**. Risk of outcomes (1 day to 5 years)_exclude those medicine not approval for RA

| **Outcomes** | **Patients with outcome** | | **Hazard ratio**  **(95% CI) ^a^** |
| --- | --- | --- | --- |
|  | RZV cohort | Control cohort |  |
| **Herpes zoster** | 651 | 664 | **0.873 (0.784-0.973)** |
| Zoster encephalitis | 10 | 10 | 0.887 (0.179-4.395) |
| Zoster meningitis | 10 | 10 | 0.149 (0.018-1.238) |
| Zoster with other nervous system involvement | 151 | 134 | 1.008 (0.799-1.272) |
| Zoster ocular disease | 39 | 50 | 0.697 (0.459-1.060) |
| Disseminated zoster | 19 | 19 | 0.899 (0.476-1.697) |
| Zoster with other complications | 95 | 90 | 0.936 (0.701-1.249) |
| Zoster without complications | 520 | 532 | **0.870 (0.771-0.982)** |
| **All-cause mortality** | 1453 | 2075 | **0.623 (0.582-0.666)^*^** |

Note:

RZV: Recombinant zoster vaccine, CI: Confidence interval. NA: Not available.

If the patient is less or equal to 10, results show the count as 10.

1. Propensity score matching was performed on age at index, sex, race, SES, lifestyles, medical utilization, comorbidities and corticosteroids.

* Proportionality <0.001.

**Supplementary Table 5**. Risk of outcomes (1 day to 5 years)_2 doses vs. 1 dose

| **Outcomes** | **Patients with outcome** | | **Hazard ratio**  **(95% CI) ^a^** |
| --- | --- | --- | --- |
|  | 2 doses RZV cohort ^b^  (n=8719) | 1 dose RZV cohort ^c^  (n=8719) |  |
| **Herpes zoster** | 218 | 208 | 0.929 (0.769-1.124) |
| Zoster encephalitis | 10 | 10 | 1.827 (0.165-20.19) |
| Zoster meningitis | 10 | 0 | NA |
| Zoster with other nervous system involvement | 39 | 52 | 0.668 (0.441-1.012) |
| Zoster ocular disease | 18 | 10 | 1.565 (0.722-3.391) |
| Disseminated zoster | 10 | 10 | 1.586 (0.464-5.423) |
| Zoster with other complications | 18 | 33 | **0.481 (0.271-0.855)** |
| Zoster without complications | 180 | 158 | 1.013 (0.818-1.254) |
| **All-cause mortality** | 477 | 651 | **0.635 (0.565-0.715)^*^** |

Note:

RZV: Recombinant zoster vaccine, CI: Confidence interval. NA: Not available.

If the patient is less or equal to 10, results show the count as 10.

1. Propensity score matching was performed on age at index, sex, race, SES, lifestyles, medical utilization, comorbidities and corticosteroids.
2. Received 2^nd^ dose within 2 months and 1 year after the first instance of RZV vaccination.
3. Never received other RZV vaccine at least 1 day after the first instance of RZV vaccination.

* Proportionality <0.001.

**Supplementary Table 6**. Risk of outcomes (1 day to 5 years)_ RZV in RA patients vs. RZV in non-RA subjects

| **Outcomes** | **Patients with outcome** | | **Hazard ratio**  **(95% CI) ^a^** |
| --- | --- | --- | --- |
|  | RZV in RA  cohort  (n=23763) | RZV in non-RA  cohort  (n=23763) |  |
| **Herpes zoster** | 601 | 283 | **2.033 (1.765-2.342)** |
| Zoster encephalitis | 10 | 0 | NA |
| Zoster meningitis | 10 | 0 | NA |
| Zoster with other nervous system involvement | 145 | 53 | **2.608 (1.904-3.573)** |
| Zoster ocular disease | 33 | 21 | 1.490 (0.862-2.575) |
| Disseminated zoster | 17 | 10 | **2.714 (1.070-6.884)** |
| Zoster with other complications | 89 | 31 | **2.721 (1.808-4.095)** |
| Zoster without complications | 476 | 224 | **2.031 (1.732-2.380)** |
| **All-cause mortality** | 1360 | 1504 | **0.853 (0.793-0.918)** |

Note:

RA: Rheumatoid arthritis, RZV: Recombinant zoster vaccine, CI: Confidence interval. NA: Not available.

If the patient is less or equal to 10, results show the count as 10.

1. Propensity score matching was performed on age at index, sex, race, SES, lifestyles, medical utilization, comorbidities and corticosteroids.

**Supplementary Table 7**. Risk of outcomes_ stratified by age at index

| **Outcomes**  **(**RZV cohort vs. Control cohort) | **Hazard ratio (95% CI) ^a^** | |
| --- | --- | --- |
|  | 50~64y  (n=10974 pairs) | ≧ 65y  (n=10470 pairs) |
| **Herpes zoster** | **0.731 (0.610-0.876)** | 0.921 (0.781-1.087) |
| Zoster encephalitis | 1.003 (0.063-16.07) | 1.803 (0.163-19.89) |
| Zoster meningitis | NA | NA |
| Zoster with other nervous system involvement | 0.871 (0.579-1.310) | 0.983 (0.723-1.336) |
| Zoster ocular disease | 1.003 (0.483-2.082) | 1.105 (0.561-2.175) |
| Disseminated zoster | 0.474 (0.164-1.369) | 1.003 (0.337-2.988) |
| Zoster with other complications | 0.748 (0.440-1.271) | 1.156 (0.709-1.884) |
| Zoster without complications | **0.698 (0.569-0.855)** | 0.928 (0.770-1.118) |
| **All-cause mortality** | **0.578 (0.501-0.668)** | **0.637 (0.581-0.698)^*^** |

Note:

RZV: Recombinant zoster vaccine, CI: Confidence interval. NA: Not available.

a. Propensity score matching was performed on age at index, sex, race, SES, lifestyles, medical utilization, comorbidities and corticosteroids.

* Proportionality <0.001.

**Supplementary Table 8**. Risk of outcomes_ stratified by sex

| **Outcomes**  **(**RZV cohort vs. Control cohort) | **Hazard ratio (95% CI) ^a^** | |
| --- | --- | --- |
|  | Male  (n= 4964 pairs) | Female  (n= 14923 pairs) |
| **Herpes zoster** | 0.784 (0.587-1.046) | **0.791 (0.684-0.915)** |
| Zoster encephalitis | NA | NA |
| Zoster meningitis | NA | NA |
| Zoster with other nervous system involvement | 0.844 (0.427-1.672) | 0.854 (0.641-1.136) |
| Zoster ocular disease | 1.636 (0.548-4.885) | 0.741 (0.408-1.347) |
| Disseminated zoster | 1.522 (0.363-6.370) | 1.101 (0.370-3.277) |
| Zoster with other complications | 1.025 (0.488-2.154) | 0.691 (0.460-1.039) |
| Zoster without complications | 0.754 (0.545-1.044) | **0.789 (0.669-0.930)** |
| **All-cause mortality** | **0.601 (0.524-0.689)^*^** | **0.632 (0.572-0.698)** |

Note:

RZV: Recombinant zoster vaccine, CI: Confidence interval. NA: Not available.

a. Propensity score matching was performed on age at index, sex, race, SES, lifestyles, medical utilization, comorbidities and corticosteroids.

* Proportionality <0.001.

**Supplementary Table 9**. Risk of outcomes_ stratified by race

| **Outcomes**  **(**RZV cohort vs. Control cohort) | **Hazard ratio (95% CI) ^a^** | |
| --- | --- | --- |
|  | White  (n= 14479 pairs) | Black or African American  (n= 2964 pairs) |
| **Herpes zoster** | **0.808 (0.696-0.937)** | **0.685 (0.481-0.976)** |
| Zoster encephalitis | 0.600 (0.100-3.594) | NA |
| Zoster meningitis | 1.020 (0.064-16.33) | NA |
| Zoster with other nervous system involvement | 0.918 (0.670-1.258) | 0.765 (0.383-1.528) |
| Zoster ocular disease | 0.749 (0.416-1.350) | 0.513 (0.157-1.673) |
| Disseminated zoster | 1.577 (0.462-5.388) | 4.714 (0.522-42.59) |
| Zoster with other complications | 0.707 (0.470-1.063) | 1.774 (0.644-4.888) |
| Zoster without complications | **0.791 (0.671-0.933)** | **0.641 (0.423-0.971)** |
| **All-cause mortality** | **0.642 (0.587-0.703)^*^** | **0.496 (0.394-0.624)** |

Note:

RZV: Recombinant zoster vaccine, CI: Confidence interval. NA: Not available.

a. Propensity score matching was performed on age at index, sex, race, SES, lifestyles, medical utilization, comorbidities and corticosteroids.

* Proportionality <0.001.
