# Supplementary material for: Effectiveness of recombinant zoster vaccine in reducing herpes zoster incidence and all-cause mortality among patients with rheumatoid arthritis: a retrospective cohort study of 21,046 individuals from TriNetX U.S. Collaborative Network
Source: eClinicalMedicine. 2025 Jun 25;85:103319. doi: 10.1016/j.eclinm.2025.103319 (PMC12246860; doi:10.1016/j.eclinm.2025.103319)
Supplement: Captions for Supplementary Materials [file mmc2.docx]

**Supplementary Table 1.** Codes utilized in cohort identification

The table presents National Library of Medicine (NLM) codes used to identify **rheumatoid arthritis medications** in the study cohort, including codes from the Veterans Affairs Drug Classification System and RXNORM normalized naming system for **disease-modifying antirheumatic drugs (DMARDs), biologics, and immunosuppressants.**

**Supplementary Table 2.** Risk of outcomes_ different follow up duration

This table compares the risk of outcomes between the recombinant zoster vaccine (RZV) cohort and the control cohort across varying follow-up durations (from 1 day to 1 year, 2 years, 3 years, and 4 years). Hazard ratios (HRs) with 95% confidence intervals (CIs) are presented for herpes zoster and its manifestations, as well as all-cause mortality. Propensity score matching was performed on age at index, sex, race, socioeconomic status, lifestyles, medical utilization, comorbidities, and corticosteroid use.

**Supplementary Table 3.** Risk of outcomes_ matching with different variables

This table presents the risk of outcomes comparing the recombinant zoster vaccine (RZV) cohort to the control cohort using different propensity score matching models. Model 1 represents the crude analysis before matching. Model 2 includes matching on age, sex, race, socioeconomic status, lifestyles, and medical utilization. Model 3 (the main model) additionally includes comorbidities and corticosteroid use. Model 4 incorporates all listed variables.

**Supplementary Table 4.** Risk of outcomes (1 day to 5 years)_exclude those medicine not approval for RA

This table presents the risk of outcomes over a 1-day to 5-year follow-up period, excluding patients using medications not approved for rheumatoid arthritis treatment. The analysis compares the RZV cohort to the control cohort in terms of herpes zoster outcomes and all-cause mortality. Propensity score matching was performed on age at index, sex, race, socioeconomic status, lifestyles, medical utilization, comorbidities, and corticosteroid use.

**Supplementary Table 5.** Risk of outcomes (1 day to 5 years)_2 doses vs. 1 dose

This table presents the risk of outcomes over a 1-day to 5-year follow-up period, comparing patients who received two doses versus one dose of the recombinant zoster vaccine (RZV). The two-dose cohort received the second dose within 2 months to 1 year after the first vaccination, while the one-dose cohort did not receive an additional RZV dose at any time ≥1 day after the first dose. Propensity score matching was performed on age at index, sex, race, socioeconomic status, lifestyles, medical utilization, comorbidities, and corticosteroid use.

**Supplementary Table 6.** Risk of outcomes (1 day to 5 years)_ RZV in RA patients vs. RZV in non-RA subjects

This table presents the risk of outcomes over a 1-day to 5-year follow-up period, comparing the effectiveness of the recombinant zoster vaccine (RZV) in patients with rheumatoid arthritis (RA) versus individuals without RA. The analysis highlights the differential risk of herpes zoster outcomes and mortality between these two populations following RZV vaccination. Propensity score matching was performed on age at index, sex, race, socioeconomic status, lifestyles, medical utilization, comorbidities, and corticosteroid use.

**Supplementary Table 7.** Risk of outcomes_ stratified by age at index

This table presents the risk of outcomes stratified by age at index, comparing the RZV cohort to the control cohort across two age groups: 50–64 years and ≥65 years. Hazard ratios (HRs) with 95% confidence intervals (CIs) are reported for herpes zoster outcomes and all-cause mortality. Propensity score matching was performed on age at index, sex, race, socioeconomic status, lifestyles, medical utilization, comorbidities, and corticosteroid use.

**Supplementary Table 8.** Risk of outcomes_ stratified by sex

This table presents the risk of outcomes stratified by sex, comparing the RZV cohort to the control cohort in male and female populations. Hazard ratios (HRs) **with 95% confidence intervals (CIs)** are reported for herpes zoster outcomes and all-cause mortality by sex. Propensity score matching was performed on age at index, sex, race, socioeconomic status, lifestyles, medical utilization, comorbidities, and corticosteroid use.

**Supplementary Table 9.** Risk of outcomes_ stratified by race

This table presents the risk of outcomes stratified by race, comparing the RZV cohort to the control cohort in White and Black or African American populations. Hazard ratios (HRs) with 95% confidence intervals (CIs) are reported for herpes zoster outcomes and all-cause mortality by racial group. Propensity score matching was performed on age at index, sex, race, socioeconomic status, lifestyles, medical utilization, comorbidities, and corticosteroid use.
